# Supplementary material for: Single-cell spatial multi-omics and deep learning dissect enhancer-driven gene regulatory networks in liver zonation
Source: Nat Cell Biol. 2024 Jan 5;26(1):153–67. doi: 10.1038/s41556-023-01316-4 (PMC10791584; doi:10.1038/s41556-023-01316-4)
Supplement: Supplementary file 1 — Supplementary Note and Supplementary Figs. 1–9. [file 41556_2023_1316_MOESM1_ESM.pdf]

nature cell biology

Article

https://doi.org/10.1038/s41556-023-01316-4

Single-cell spatial multi-omics and deep learning dissect enhancer-driven gene regulatory networks in liver zonation

In the format provided by the authors and unedited

Table of Contents

Supplementary Note 2
Batch effects in hepatocytes 2
Impact of sex dimorphism 3
Comparison of *in vitro* models to test hepatocyte specific enhancer regions 4
Description of single-cell data on SCope 5
Description of data at the UCSC genome browser 8
Supplementary Figures 10
References 19

Supplementary Note

Batch effects in hepatocytes

We identified an interesting batch effect in hepatocytes, related to differences in physiological state between the mice (Supplementary Fig. 1), including circadian rhythm, nutritional status, and hormone levels. For instance, Gene Ontology analysis with GREAT links two complementary topics (Topic 17 and Topic 75) to positive and negative regulation of the circadian rhythm (adjusted p-value = 10^{-19} and 10^{-7}, respectively); while four topics show enrichment for different GO terms related to hormone response and metabolism (Supplementary Fig. 2). In addition, motif enrichment analysis revealed the presence of binding sites of the circadian rhythm transcription factor CLOCK in topic 75, with a Normalized Enrichment Score (NES) of 4.25. Importantly, topic 17 is specifically enriched in hepatocytes from starved mice, while topic 75 is enriched in hepatocytes from mice fed *ad libidum*. These analyses suggest that regions specifically accessible in the two groups of samples are controlling the circadian rhythm genes. Using publicly available scRNA-seq data on the mouse liver during different phases of the circadian rythm[1], we confirmed that the animals that were fed *ad libitum* mapped to early zeitgeber timepoints, while samples for which food was removed the night before mapped to late zeitgeber timepoints (Supplementary Fig. 3, Supplementary Fig. 4, see *Methods*).

As starved samples were profiled by single cell multiomics and unstarved samples were profiled by snRNA-seq, we performed two additional single cell multiomics runs in a mouse fed *ad libidum*. Analysis with the new data again revealed two topics specific to starved (topic 72) and unstarved (topic 68) mice, with enrichment for positive and negative regulation of the circadian rhythm (adjusted p-value = 10^{-4} and 10^{-9}, respectively) based on GO analysis with GREAT; and motif enrichment analysis revealed the presence of CLOCK binding sites in the unstarved mice topic (topic 68). The new multiomics samples fed *ad libidum* also mapped to early zeitgeber points (Supplementary Fig. 5). Altogether, the circadian rhythm regulatory topics and the differential expression of circadian rhythm genes observed are driven by nutritional status, rather than experimental set-up, which suggests that the observed effect is of biological nature.

Next, we examined signaling pathways that have been reported to be affected by liver zonation, including RAS and WNT signaling, hypoxia and pituitary hormone responses[2–6]. Our analyses revealed that all these pathways change along the porto-central axis (adjusted p-value < 0.05). Particularly, hypoxia and WNT target genes are upregulated pericentrally and repressed periportally; RAS target genes are upregulated periportally and repressed pericentrally, and pituitary hormone targets are repressed pericentrally. However, hypoxia, pituitary hormones and RAS signaling target gene expression is highly variable between individuals and circadian rhythm phases (adjusted p-value < 0.05); while WNT signaling targets are consistently expressed across all samples (adjusted p-value = 0.17, Supplementary Fig. 3, Supplementary Fig. 4).

Impact of sex dimorphism

To assess whether the core hepatocyte eGRN was affected by sex dimorphism, we analyzed additional snRNA-seq data from wild-type male and female livers[7]. After quality control (see *Methods*), 4,860 liver cells from 3 different male mice and 5,342 cells from 3 different female mice were retained (Supplementary Fig. 6a-b). Importantly, neither the expression of the core hepatocyte TFs nor their targets (measured as the AUC enrichment of the SCENIC+ eRegulons) were affected by gender (Supplementary Fig. 6c-d); in other words, the expression of *Tbx3, Tcf7l1, Hnf4a, Hnf1a, Cebpa, Foxa1, Onecut1* and their targets is comparable between male and female mice.

We performed differential gene expression based on gender for all the cell types in the liver and identified 56 and 65 genes upregulated in male and female hepatocytes, respectively (LogFC > 0.75, adjusted p-value < 0.05, Supplementary Fig. 6e). Only 1 TF was upregulated in male hepatocytes, *Bcl6*, while 4 TFs were upregulated in female hepatocytes, namely *Rfx4, Cux2, Esr1 and Esrrg*. Except for *Rfx4*, these TFs have been previously reported to regulate sex differences in the mouse liver[8–10]. Gene Ontology analysis of these differentially expressed genes points to metabolic differences between male and female hepatocytes, with genes related to lipid metabolism upregulated in females (adjusted p-value: 10^{-7}, Supplementary Fig. 6f-g). Importantly, none of these genes have been found as TCF7L1 or TBX3 targets.

Altogether, this data suggests that, while there are differentially regulated genes in male and females, the core hepatocyte eGRN is not affected by sexual dimorphism. Nevertheless, additional single-cell multiomics data in female livers and/or disease models may be needed in the future to study sex-biased gene regulation in wild-type mice and upon disease at the enhancer-GRNs resolution.

Comparison of *in vitro* models to test hepatocyte specific enhancer regions

In vitro models can be used to perform MPRA experiments and more sensitive enhancer assays that are not feasible *in vivo* (i.e. luciferase assays). We compared three different hepatocyte cell lines, namely HepG2 (human hepatocellular carcinoma), Hepa1-6 (murine hepatoma), and AML12 (hepatocytes from a mouse transgenic for human TGFα). To assess their relevance as model system, we re-used RNA-seq and performed ATAC-seq on the two mouse hepatocyte cell lines (see *Methods*). At the transcriptome level, HepG2 expresses several hepatocyte master regulators from hepatocytes, such as *Hnf4a, Cebpa, Hnf1a, Tcf7l2, Foxa1,* and *Onecut1*, among others. On the other hand, AML12 shows reduced expression of *Cebpa, Foxa1, Hnf1a,* and *Hnf4a* compared to HepG2; and Hepa1-6, of *Cebpa* and *Foxa1* (Supplementary Fig. 7a). We also compared the accessibility of the 12,000 enhancers library on the different cell lines. This library included shared regions (accessible in hepatocytes and other cell types, of which 56% are promoters) and hepatocyte-specific regions (generally accessible and zonated). We observed that shared regions were largely accessible across the 3 cell lines, while HepG2 showed more accessibility in hepatocyte specific regions compared to the other cell lines (Supplementary Fig. 8a). To further assess differences at the enhancer activity level, we performed an MPRA experiment in AML12. As expected from the chromatin accessibility profiles, only positive controls and shared regions were significantly active compared to the negative control (Supplementary Fig. 7b-d). Altogether, HepG2 is more similar to *in vivo* hepatocytes in terms of TF repertoire and functionality of the enhancer library than AML12 and Hepa1-6.

Due to a mutation in beta-catenin, WNT signaling is active in HepG2[11]. It has also been reported in literature that HepG2 exhibits a more pericentral identity[12]. HepG2 expresses *Tbx3* and other pericentral markers (including *Tbx3*), while periportal genes

are not expressed or very lowly expressed, as observed in pericentral mouse hepatocytes (Supplementary Fig. 7a). Nevertheless, despite WNT activation, HepG2 does not fully recapitulate the pericentral hepatocyte identity. For instance, out of the 3,939 pericentrally (and pericentral-intermediate) zonated regions found in the mouse liver and conserved in the human genome, only 1,258 are accessible in HepG2 (Supplementary Fig. 8b).

HepG2 has been used extensively to validate mouse, human, and synthetic hepatocyte enhancers[13–17]. For instance, Smith et al. (2013)[17] performed MPRA experiments in the mouse liver and HepG2 using a synthetic library of enhancer formed by combinations of binding sites of 12 hepatocyte TFs (AHR/ARNT, CEBPA, FOXA1, GATA4, HNF1A, HNF4A, NR2F2, ONECUT1, PPARA, RXRA, TFAP2C and XBP1), finding a strong correlation (0.81) between the measurements in the two systems. Altogether, taking into account its limitations as an *in vitro* model systems, HepG2 can be used to study enhancers with binding sites of TFs that are conserved, which include HNF4A, FOXA1, CEBPA, ONECUT1, HNF1A and TBX3.

Description of single-cell data on SCope

All processed single-cell data is available at https://scope.aertslab.org/#/Bravo_et_al_Liver. SCope is a fast, user-friendly visualization tool for large-scale single cell data sets[18]. The following files are available at SCope:

- ATAC
  - Mouse
    - Liver-cell_gene-all-2: Loom file containing data processed with pycisTopic of the 2 snATAC-seq and 2 multiome (snATAC-seq layer) samples. The data set contains 21,393 cells and gene activities as values. In the metadata, ‘sample_id’ indicates the sample of origin, and ‘Refined_cell_type’ indicates the cell type annotation. Under coordinates, the uncorrected and batch corrected UMAPs are labelled as ‘probability_UMAP’ and ‘harmony_probability_UMAP’, respectively.
    - Cell_region-all_v2: Loom file containing data processed with pycisTopic of the 2 snATAC-seq and 2 multiome (snATAC-seq

layer) samples. The data set contains 21,393 cells, region accessibility probabilities as values, and topics as regulons. In the metadata, ‘sample_id’ indicates the sample of origin, and ‘Refined_cell_type’ indicates the cell type annotation. Under coordinates, the uncorrected and batch corrected UMAPs are labelled as ‘probability_UMAP’ and ‘harmony_probability_UMAP’, respectively.

  - Human
    - pycisTopic_gene_activity_wsignatures_v2: Loom file containing human liver data from Zhang et al. (2021)[19] processed with pycisTopic. The data set contains 6,366 cells, gene activities as values, and the SCENIC+ gene-based eRegulons inferred in the mouse liver (and converted to human symbols) as regulons. In the metadata, ‘cell_type_refined’ indicates the cell type annotation.
    - pycisTopic_region_accessibility-1: Loom file containing human liver data from Zhang et al. (2021) processed with pycisTopic. The data set contains 6,366 cells, region accessibility probabilities as values, and topics as regulons. In the metadata, ‘cell_type_refined’ indicates the cell type annotation.
- RNA
  - RNA+Multiome_integrated_HQ-10-1: Loom file containing data processed with VSN-pipelines of the 4 snRNA-seq and 2 multiome (snRNA-seq layer) samples. The data set contains 29,798 cells and gene expression as values. In the metadata, ‘sample_id’ indicates the sample of origin, and ‘Refined_cell_type’ indicates the cell type annotation. Under coordinates, the uncorrected and batch corrected UMAPs are labelled as ‘Uncorrected UMAP’ and ‘HVG UMAP’, respectively.
  - Sex_dimorphism: Loom file containing data from male and female livers from Goldfarb et al. (2022) processed with VSN-pipelines. The data set contains 10,202 cells, gene expression as values, and the SCENIC+ gene-based eRegulons inferred in the mouse liver as regulons. In the

metadata, ‘sample_id’ indicates the sample of origin and ‘cluster_ann’ indicate the cell type annotation.

- Spatial
  - Reference_Free_ScoMAP_ATAC_MO: Loom file containing mouse liver spatial data (Resolve), in which snATAC-seq data has been projected using SCoMAP. The data set contains 14,296 cells, mapped region accessibility probabilities as values a mapped topics as regulons. In the metadata, ‘Cell type’ indicates the cell type annotation.
  - Reference_Free_ScoMAP_RNA_MO: Loom file containing mouse liver spatial data (Resolve), in which snRNA-seq data has been projected using SCoMAP. The data set contains 14,296 cells and mapped gene expression as values. In the metadata, ‘Cell type’ indicates the cell type annotation.
  - Resolve_Liver: Loom file containing mouse liver spatial data (Resolve), with the measured gene expression values (for 100 genes). The data set contains 14,296 cells and gene expression as values. In the metadata, ‘sample_id’ indicates the sample of origin and ‘cell_type’ indicates the cell type annotation.
  - SCENIC+_gene_based_ScoMAP_RNA_MO: Loom file containing SCoMAP liver lobule template, in which snRNA-seq data has been projected. The data set contains 4,498 cells, gene expression as values, and SCENIC+ gene-based eRegulons inferred in the mouse liver as regulons. In the metadata, ‘Cell type’ indicates the cell type annotation.
  - ScoMAP_ATAC_MO: Loom file containing SCoMAP liver lobule template, in which snATAC-seq data has been projected. The data set contains 4,498 cells, region accessibility probabilities as values, and topics as regulons. In the metadata, ‘Cell type’ indicates the cell type annotation.
- SCENIC+
  - SCENIC+_curated_gene_based-3: Loom file containing data processed with VSN-pipelines of the 4 snRNA-seq and 2 multiome (snRNA-seq layer) samples. The data set contains 29,798 cells, gene expression as values, and the SCENIC+ gene-based eRegulons inferred in the mouse

liver as regulons. In the metadata, 'sample_id' indicates the sample of origin, and 'Refined_cell_type' indicates the cell type annotation. Under coordinates, the uncorrected and batch corrected UMAPs are labelled as 'Uncorrected UMAP' and 'HVG UMAP', respectively.

    - SCENIC+_curated_region_based: Loom file containing data processed with pycisTopic of the 2 snATAC-seq and 2 multiome (snATAC-seq layer) samples. The data set contains 21,393 cells, region accessibility probabilities as values, and the SCENIC+ region-based eRegulons inferred in the mouse liver as regulons. In the metadata, 'sample_id' indicates the sample of origin, and 'Refined_cell_type' indicates the cell type annotation. Under coordinates, the uncorrected and batch corrected UMAPs are labelled as 'probability_UMAP' and 'harmony_probability_UMAP', respectively.

Description of data at the UCSC genome browser

Processed genomic data can be explored at https://genome.ucsc.edu/s/cbravo/Bravo_et_al_Liver. The following files are available at the UCSC genome browser session:

- Custom tracks: Bed files containing mouse liver accessible regions identified from snATAC-seq and multiome data (486,888 regions, 'Consensus peaks'), shared hepatocyte regions (14,005 regions, 'Shared hepatocyte regions'), periportal shared regions (1,324 regions, 'Periportal shared'), intermediate shared regions (3,511 regions, 'Intermediate shared'), pericentral shared regions (1,495 regions, 'Pericentral shared') and general shared regions (7,675 regions, 'General shared') and mouse liver regions included in the CHEQ-seq library (10,845 regions).
- Bravo *et al.* Liver hub
    - Gene Expression: Bar charts showing gene expression levels per cell type.
    - ATAC-profile Celltype-ATAC: Pseudobulk bigwig files based on the ATAC-seq based cell type annotation.
    - ATAC-profile Celltype-RNA: Pseudobulk bigwig files based on the RNA-seq based cell type annotation.

- ATAC-profile Celltype-ATACpersample: Pseudobulk bigwig files based on the ATAC-seq based cell type annotation and sample of origin.
- ATAC-profile Non-parenchymal-zonation: Pseudobulk bigwig files based on zonation subtypes for LSEC and HSC
- SCENIC+ eRegulons: Bigbed file containing regions in each SCENIC+ regulon
- SCENIC+ eRegulons (selected): Bigbed file containing regions in selected hepatocyte SCENIC+ regulons
- SCENIC+ R2G: Region-to-gene links inferred by SCENIC+
- SCENIC+ R2G (eGRN): Region-to-gene links kept in the eRegulons inferred by SCENIC+
- Mouse Liver ChIP-seq: Bigwig files of mouse liver TF ChIP-seq profiles (TBX3, HNF4A, ONECUT1, CEBPA and FOXA1)
- FACS ATAC-seq: Bigwig profiles of the FAC-sorted hepatocyte fractions

Supplementary Figures

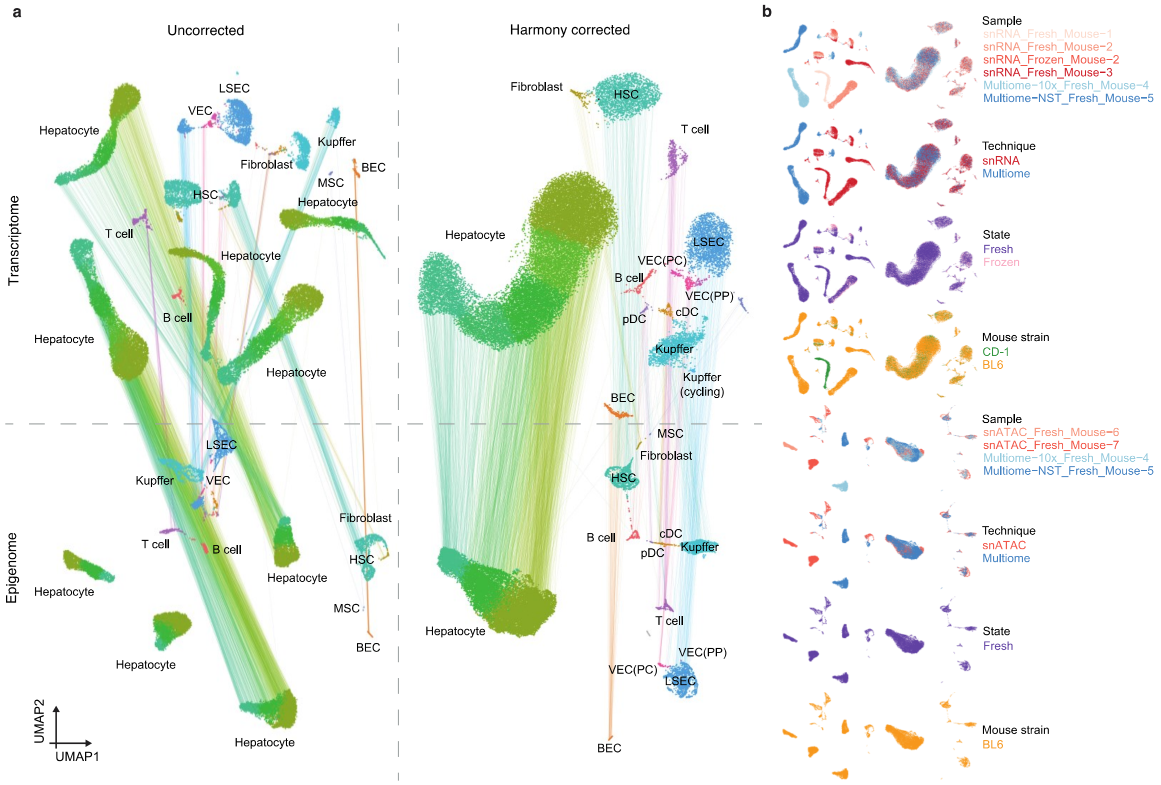

Supplementary Figure 1. Batch effects in the mouse liver. a. Transcriptome and epigenome based UMAPs (29,798 and 22,600 cells, respectively) before and after batch correction with harmony. Lines linking the UMAPs map the transcriptome and the epigenome UMAP positions from the same cell (profiled by single-cell multiomics). **b.** Uncorrected and harmony corrected transcriptome and epigenome UMAP colored by (from top to bottom) sample of origin, technique (multiome or independent snRNA-seq/snATAC-seq), sample condition (fresh/frozen) and mouse strain (CD-1, BL6). For the transcriptome and epigenome data, cells from 5 and 4 biological replicates were combined, respectively. BEC: biliary epithelial cells, cDC: conventional dendritic cell, HSC: hepatic stellate cells, LSEC: liver sinusoidal endothelial cells, MSC: mesothelial cells, PC: pericentral, pDC: plasmacytoid dendritic cell, PP: periportal, VEC: vascular endothelial cells. Source numerical data are available in FigShare (DOI: 10.6084/m9.figshare.24532951).

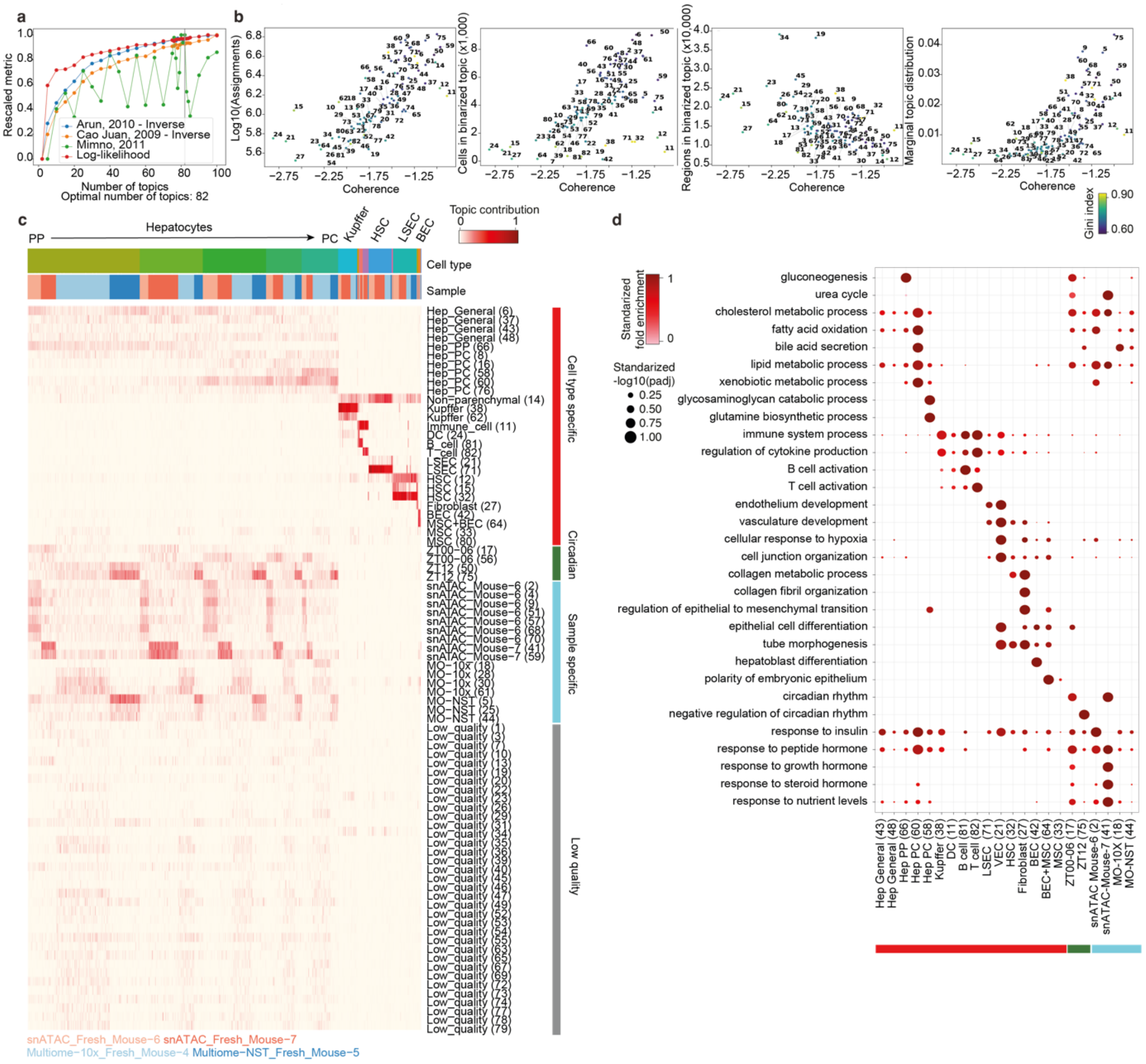

Supplementary Figure 2. Overview of regulatory topics inferred with pycisTopic from the epigenome layer. a. Standardized pycisTopic topic model selection metrics, including loglikelihood, coherence (Minmo et al. 2011), reversed Cao Juan et al., 2009 and reversed Arun, 2010. The selected topic model (82 topics) is indicated with a vertical grey line. **b.** pycisTopic topic quality metrics including the number of assignments, number of regions/cells in the binarized topics and marginal topic distribution, colored by gini index value. **c.** Cell-topic heatmap (22,600 cells) showing topic contributions across cells with annotated topics. **d.** Dotplot showing relevant GO terms found by GREAT in the topics, using the -log10(padj) as size and the standardized fold enrichment as color. The adjusted p-values are derived from the binomial test over genomic regions performed by GREAT, followed by Bonferroni correction. Cells from 4 biological replicates were combined. BEC: biliary epithelial cells, cDC: conventional dendritic cell, HSC: hepatic stellate cells, LSEC: liver sinusoidal endothelial cells, MSC: mesothelial cells, PC: pericentral, pDC: plasmacytoid dendritic cell, PP: periportal, VEC: vascular endothelial cells. Source numerical data are available in FigShare (DOI: 10.6084/m9.figshare.24532951).

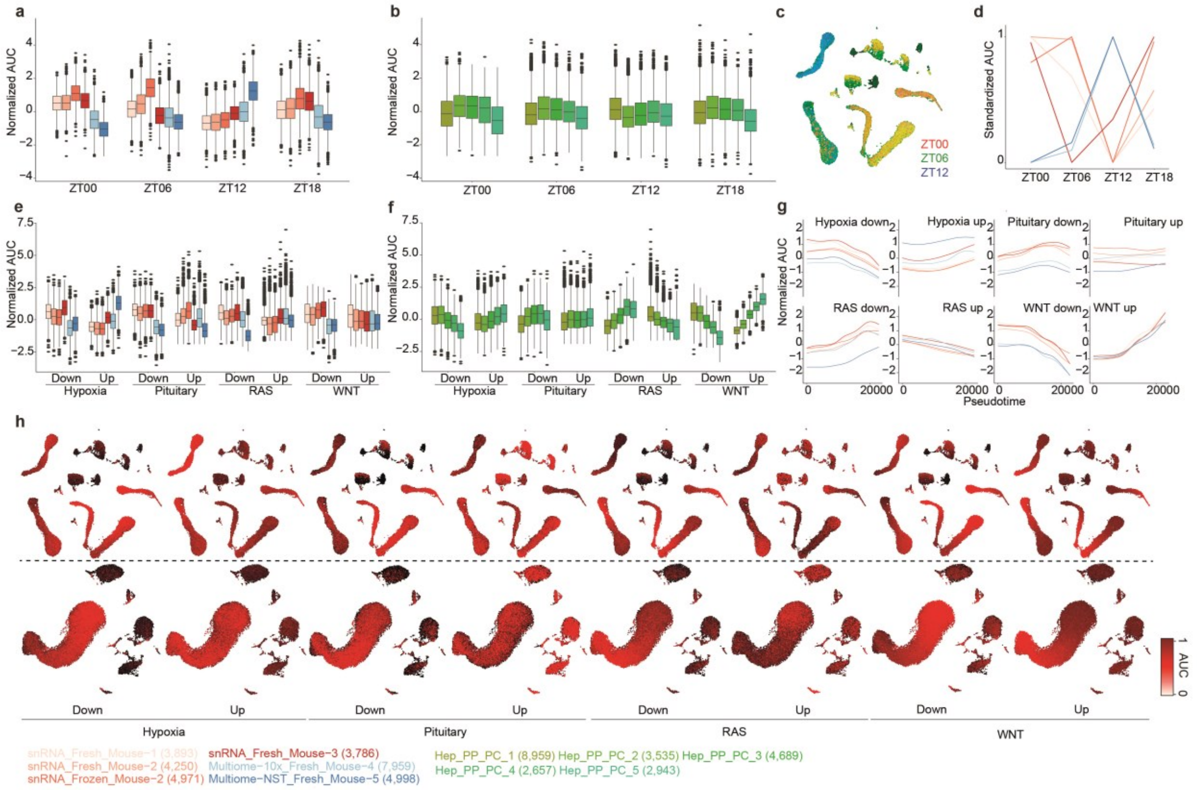

Supplementary Figure 3. Enrichment of relevant signatures in the mouse liver on the transcriptome layer. a. Boxplot showing normalized AUC values across samples for circadian rhythm signatures (genes upregulated at different Zeitgeber times (ZT)), derived from Droin et al. 2021. **b.** Boxplot showing normalized AUC values across hepatocyte subclasses (driven by zonation) for circadian rhythm signatures. **c.** Uncorrected snRNA-seq UMAP (29,798 cells) colored by AUC values for circadian rhythm signatures using RGB encoding. **d.** Standardized AUC values across samples for signatures on different circadian rhythm time points. **e.** Boxplot showing normalized AUC values across samples for signatures from different signaling pathways affected by liver zonation, derived from Halpern et al. 2017. **f.** Boxplot showing normalized AUC values across hepatocyte subclasses (driven by zonation) for signaling pathways signatures. **g.** GAM fitted eGRN AUC profiles per sample for signaling pathway signatures along the zonation pseudotime. **h.** Uncorrected (top) and corrected (bottom) UMAP colored by AUC scores for signaling pathway signatures. Cells from 5 biological replicates were combined. The number of cells per experiment (panels a, d, e and g) and per zonation group (b and f) is indicated in the legends at the bottom. In a, b, e and f, the top/lower hinge represents the upper/lower quartile and whiskers extend from the hinge to the largest/smallest value no further than 1.5 × interquartile range from the hinge, respectively. The median is used as the center. Source numerical data are available in FigShare (DOI: 10.6084/m9.figshare.24532951).

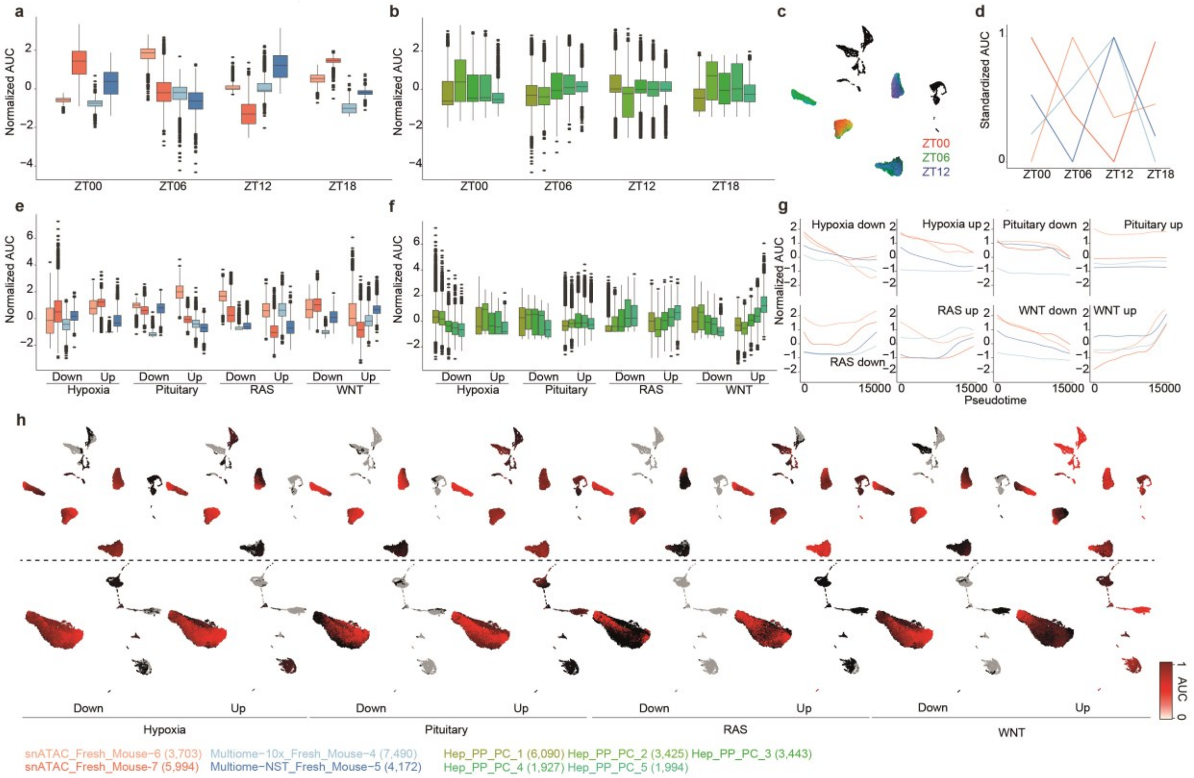

Supplementary Figure 4. Enrichment of relevant signatures in the mouse liver on the epigenome layer, relying on gene activity. a. Boxplot showing normalized AUC values across samples for circadian rhythm signatures (genes upregulated at different Zeitgeber times (ZT), derived from Droin et al. 2021. **b.** Boxplot showing normalized AUC values across hepatocyte subclasses (driven by zonation) for circadian rhythm signatures. **c.** Uncorrected snATAC-seq UMAP (22,600 cells) colored by AUC values for circadian rhythm signatures using RGB encoding. **d.** Standardized AUC values across samples for signatures on different circadian rhythm time points. **e.** Boxplot showing normalized AUC values across samples for signatures from different signaling pathways affected by liver zonation, obtained from Halpern et al. 2017. **f.** Boxplot showing normalized AUC values across hepatocyte subclasses (driven by zonation) for signaling pathways signatures. **g.** GAM fitted eGRN AUC profiles per sample for signaling pathway signatures along the zonation pseudotime. **h.** Uncorrected (top) and corrected (bottom) UMAP colored by AUC scores for signaling pathway signatures. Cells from 4 biological replicates were combined. The number of cells per experiment (panels a, d, e and g) and per zonation group (b and f) is indicated in the legends at the bottom. In a, b, e and f, the top/lower hinge represents the upper/lower quartile and whiskers extend from the hinge to the largest/smallest value no further than 1.5 × interquartile range from the hinge, respectively. The median is used as the center. Source numerical data are available in FigShare (DOI: 10.6084/m9.figshare.24532951).

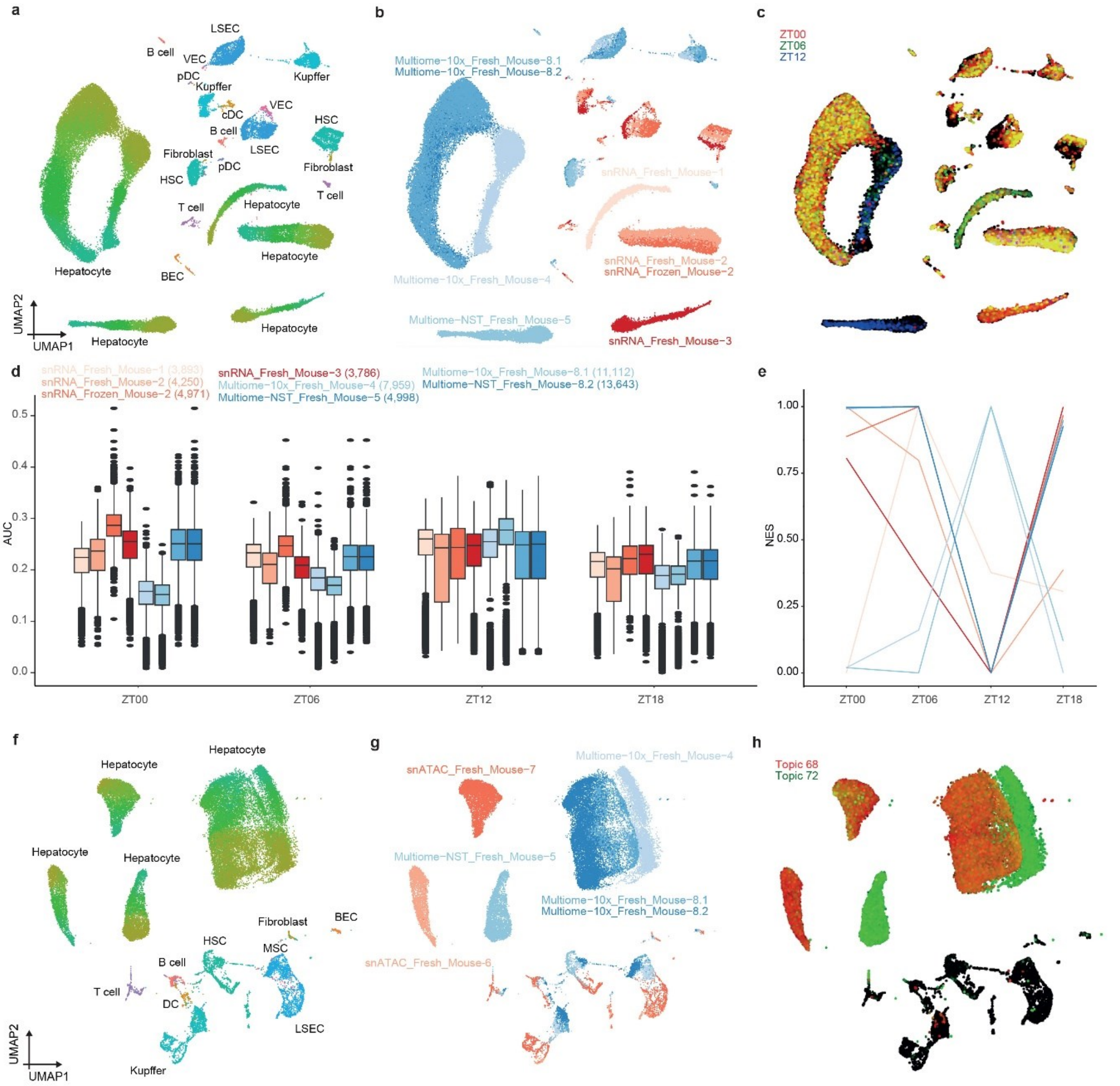

Supplementary Figure 5. Validation of circadian rhythm signatures with additional single-cell multiomics data. a. Uncorrected snRNA-seq-based UMAP of 54,612 cells profiled by snRNA-seq or multiome (snRNA-seq+snATAC-seq) colored by cell type. **b.** Uncorrected snRNA-seq-based UMAP colored by sample (technique and mouse). **c.** Uncorrected snRNA-seq-based UMAP colored by AUC values for circadian rhythm signatures (Droin et al., 2021) using RGB encoding. **d.** Boxplot showing normalized AUC values across samples for circadian rhythm signatures (genes upregulated at different Zeitgeber times (ZT). The number of cells per sample group is indicated at the top. In a, b, e and f, the top/lower hinge represents the upper/lower quartile and whiskers extend from the hinge to the largest/smallest value no further than 1.5 × interquartile range from the hinge, respectively. The median is used as the center. **e.** Standardized AUC values across samples for signatures on different circadian rhythm time points. **f.** Uncorrected snATAC-seq-based UMAP of 36,721 cells profiled by snATAC-seq or multiome (snRNA-seq+snATAC-seq) colored by cell type. **g.** Uncorrected snATAC-seq-based UMAP colored by sample (technique and mouse). **h.** Uncorrected snATAC-seq-based UMAP colored by topic probability using RGB encoding. For the transcriptome and epigenome data, cells from 6 and 5 biological replicates were combined. BEC: biliary epithelial cells, cDC: conventional dendritic cell, HSC: hepatic stellate cells, LSEC: liver sinusoidal endothelial cells, MSC: mesothelial cells, PC: pericentral, pDC: plasmacytoid dendritic cell, PP: periportal, VEC: vascular endothelial cells. Source numerical data are available in FigShare (DOI: 10.6084/m9.figshare.24532951).

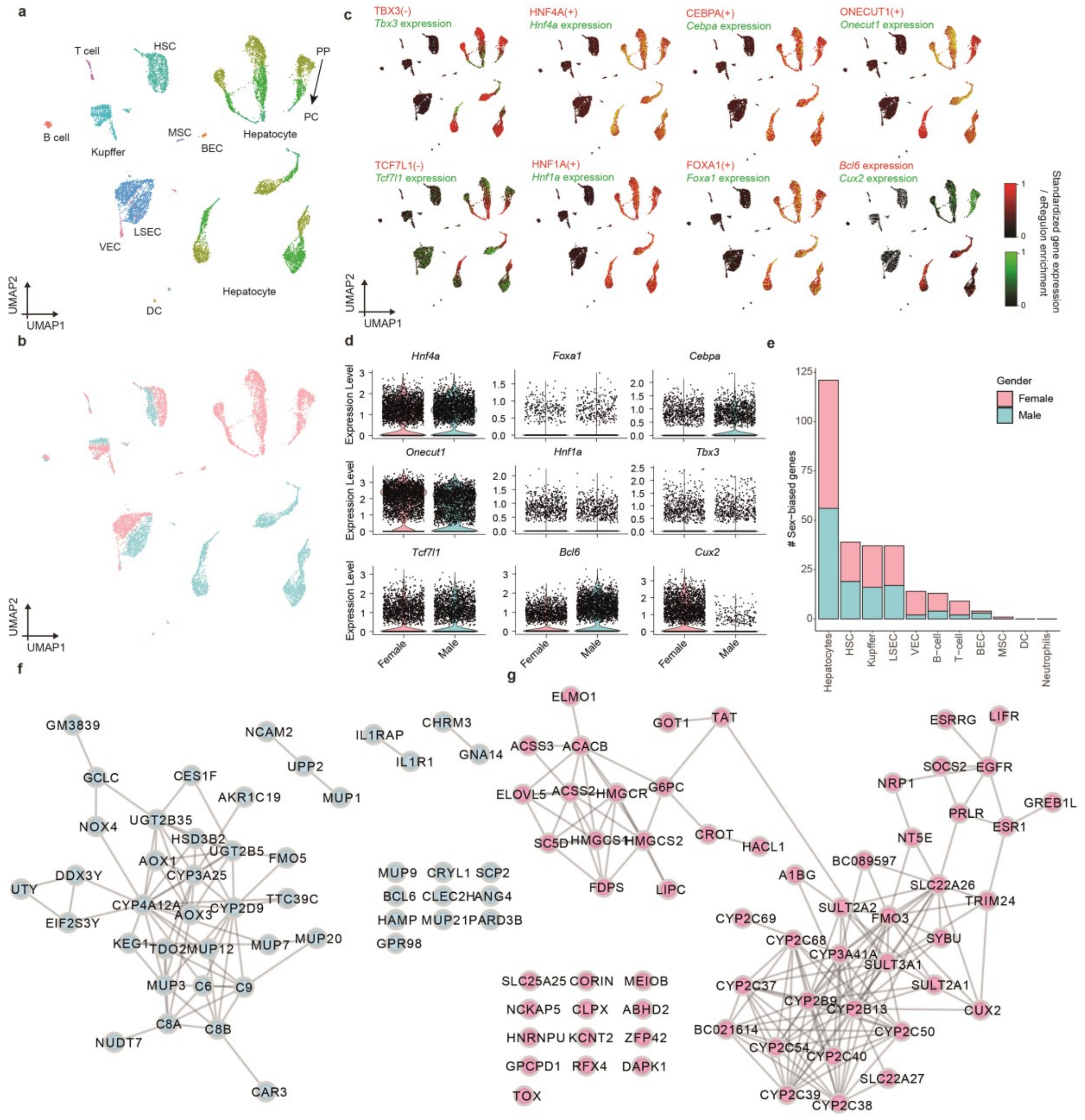

Supplementary Figure 6. Impact of sex dimorphism in gene expression in the mouse liver. **a.-b.** UMAP of 4,860 liver cells from 3 different male mice and 5,342 cells from 3 different female mice colored by cell type (**a.**) and by gender (**b.**, male: blue, female: pink)**. c.** UMAP from male and female livers colored by gene expression or eRegulon enrichment. **d**. Normalised gene expression distribution across female and male hepatocytes for selected genes**. e.** Barplot reporting the number of differentially expressed genes between male and female livers across cell types **f.-g.** STRING network based on the genes upregulated in male (**f.**) and female (**g.**) hepatocytes. BEC: biliary epithelial cells, DC: dendritic cell, HSC: hepatic stellate cells, LSEC: liver sinusoidal endothelial cells, MSC: mesothelial cells, PC: pericentral, PP: periportal, VEC: vascular endothelial cells. Source numerical data are available in FigShare (DOI: 10.6084/m9.figshare.24532951).

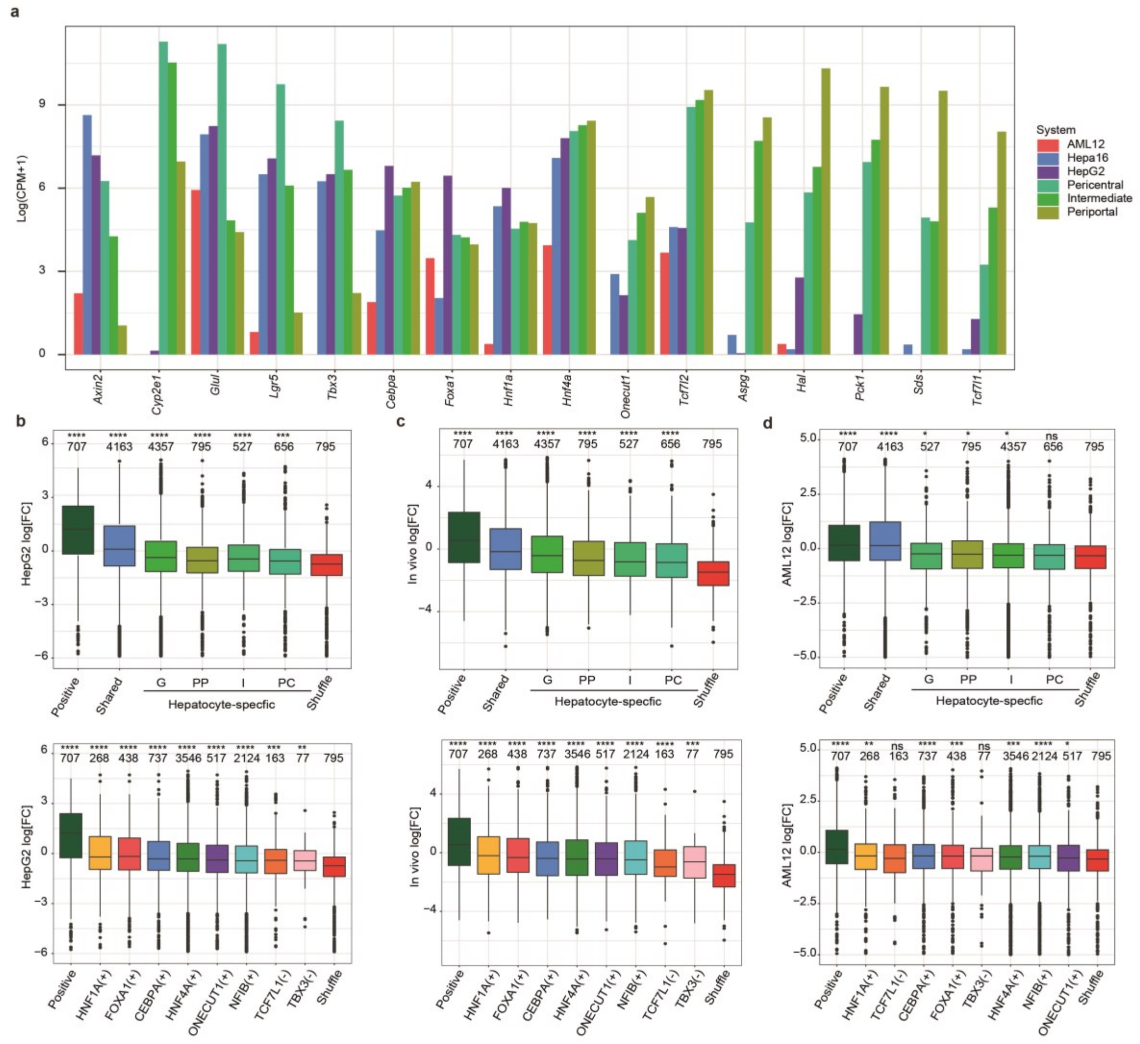

Supplementary Figure 7. Comparison of HepG2, AML12 and Hepa1-6 based on gene expression and enhancer activity. **a.** Normalized gene expression of selected genes across systems, namely AML12, Hepa1-6, HepG2 and snRNA-seq pseudolbulks of pericentral, intermediate and periportal hepatocytes**.** Transcription factors are highlighted in bold. **b.-d.** MPRA Log2 Fold-Change boxplots per enhancer class (top) and eRegulon (bottom) in HepG2 (**b.**), in vivo (**c.**), and in AML12 (**d.**). The asterisks indicate the significance compared to shuffle (****: p-value <= 0.0001, ***: p-value <= 0.001, **: p-value <= 0.01, *: p-value <= 0.05, ns: not significant). G: General, PP: Periportal, I: Intermediate, PC: Pericentral. In the boxplots in b, c and d, the top/lower hinge represents the upper/lower quartile and whiskers extend from the hinge to the largest/smallest value no further than 1.5 × interquartile range from the hinge, respectively. The median is used as the center. One-sided Wilcoxon tests were performed to assess if the LogFC values of each group were greater than those of the shuffled regions. The asterisks represent the Bonferroni adjusted p-values of the comparisons (****: p-value <= 0.0001, ***: p-value <= 0.001, **: p-value <= 0.01, *: p-value <= 0.05, ns: p-value > 0.05). Five biological replicates were used *in vivo*, two biological replicates were performed in HepG2, and two biological replicates were performed in AML12. Source numerical data are available in FigShare (DOI: 10.6084/m9.figshare.24532951).

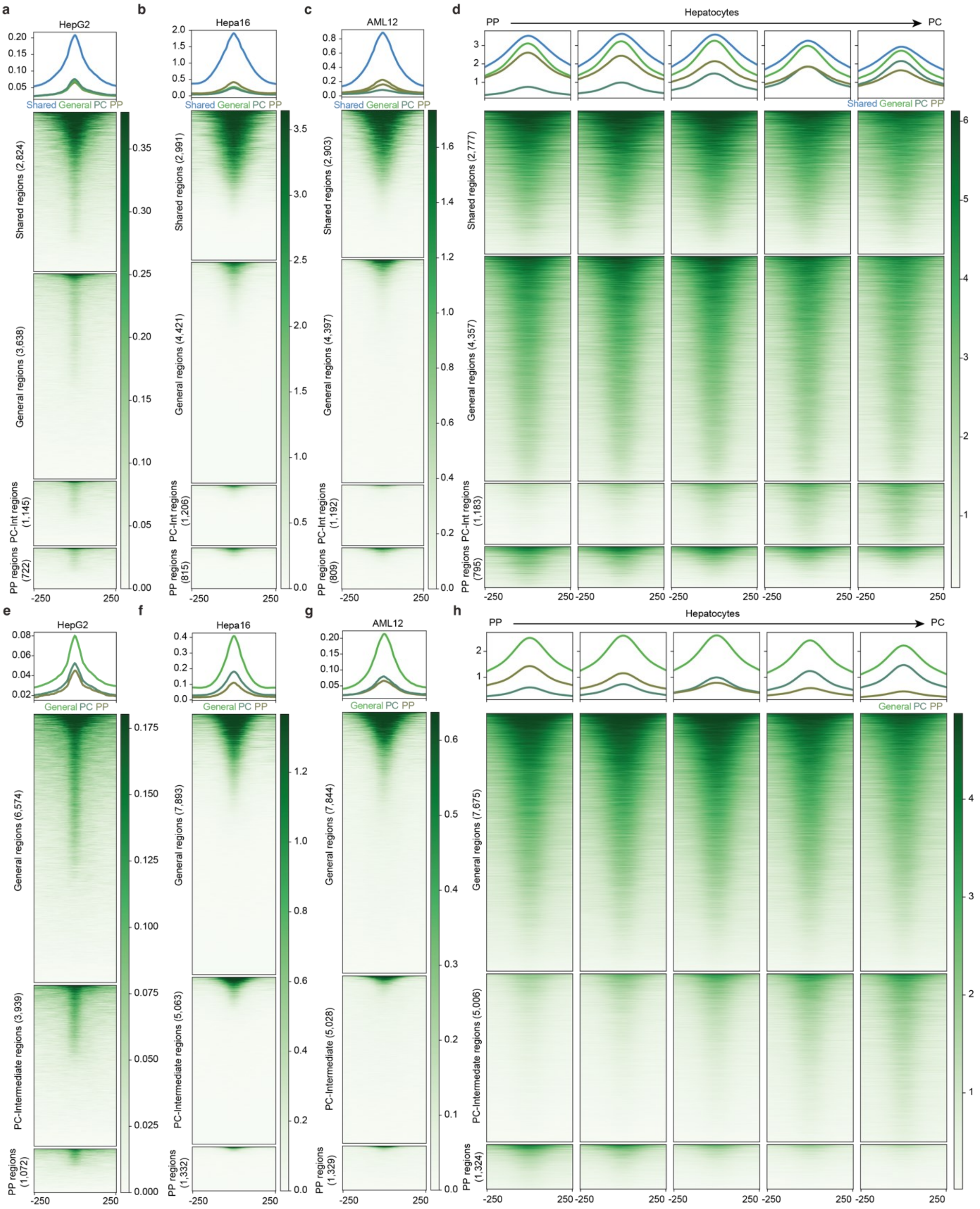

Supplementary Figure 8. Coverage of CHEQ-seq and shared hepatocyte regions across systems. a.-d. ATAC-seq coverage on the 12,000 enhancers included in the CHEQ-seq library on HepG2 (**a.**), Hepa1-6 (**b.**), AML12 (**c.**) and mouse hepatocytes pseudobulk, ordered by cluster from periportal to pericentral (**d.**). **e.-h.** ATAC-seq coverage on the shared general, pericentral and periportal regions on HepG2 (**e.**), Hepa1-6 (**f.**), AML12 (**g.**) and mouse hepatocytes pseudobulk, ordered by cluster from periportal to pericentral (**h.**). The number of regions in each group is indicated between parentheses. For the hepatocyte pseudobulks, cells from 4 biological replicates were combined.

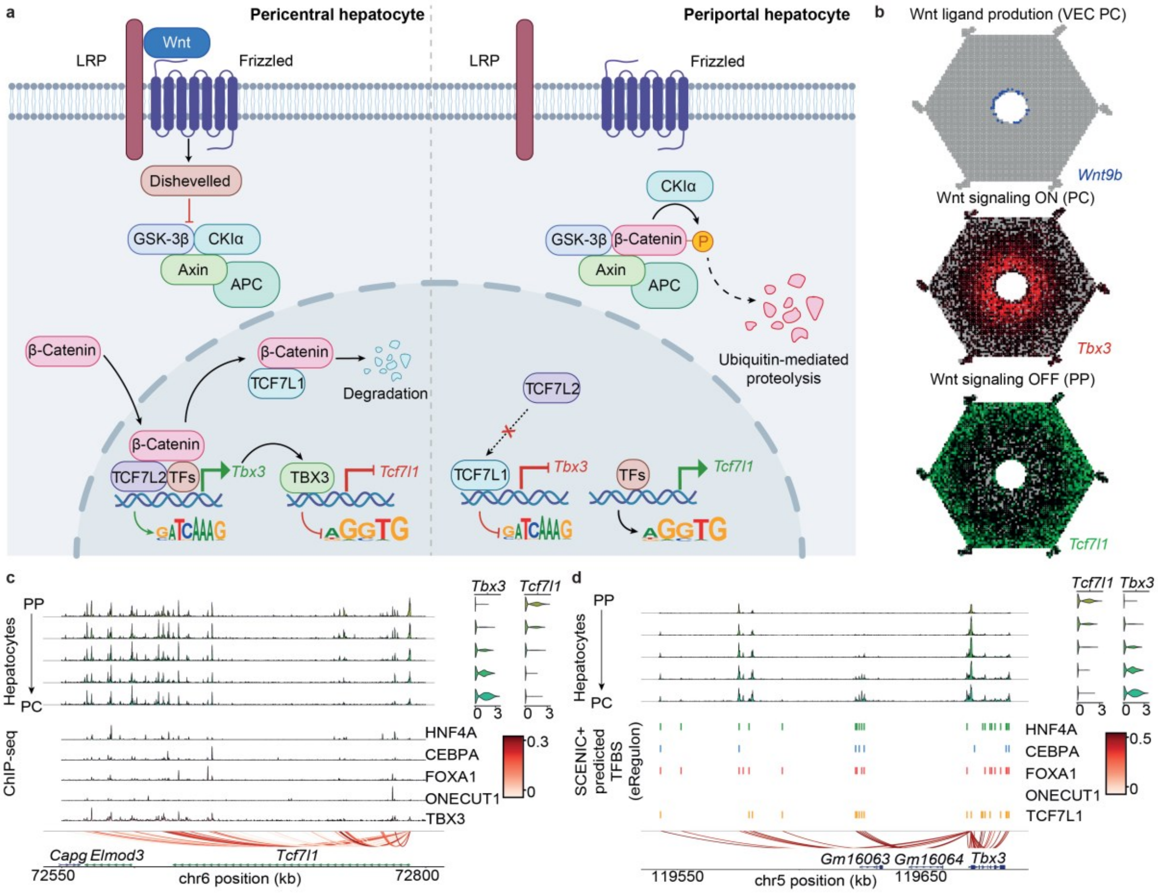

Supplementary Figure 9. Model of TCF7L1 and TBX3 roles in hepatocyte zonation. a. Schematic describing the potential regulatory function of TBX3 and TCF7L1 in hepatocyte zonation. Briefly, WNT (WNT9B, WNT2) is produced by pericentral vascular endothelial cells, creating a decreasing WNT gradient along the porto-central axis. In pericentral hepatocytes, Wnt signaling is activated resulting in the translocation of β-catenin into the nucleus. β-catenin promotes TCF7L1 degradation, which allows the binding of general hepatocyte TFs (including TCF7L2) to the TCF7L1 repressed regions, opening up chromatin. The binding of these TFs to these regions promotes the transcription of pericentral genes, including *Tbx3* (based on SCENIC+ predictions, TF ChIP-seq for HNF4A, CEBPA, FOXA1 and ONECUT1, and literature on TBX3 activation by β-catenin). TBX3 represses its target regions in pericentral hepatocytes, resulting in the silencing of periportal genes, including *Tcf7l1* (based on SCENIC+ predictions and TBX3 ChIP-seq). In periportal hepatocytes, reached by lower amounts of WNT, β-catenin is degraded. TCF7L1 represses its target regions, resulting in the silencing of pericentral genes, including *Tbx3* (based on SCENIC+ predictions). This allows the binding of hepatocyte TFs to the TBX3 target regions, opening up chromatin and activating periportal genes. Created with BioRender.com, **b.** ScoMAP liver lobule showing localized gene expression of *Wnt9b* (WNT ligand), *Tbx3* and *Tcf7l1*, using RGB encoding. **c.** Pseudobulk accessibility profiles, ChIP-seq coverage (for HNF4A, CEBPA, FOXA1, ONECUT1 and TBX3), SCENIC+ region to gene links colored by correlation score and *Tcf7l1* and *Tbx3* expression across the zonated hepatocytes classes (from periportal (PP) to pericentral (PC)) are shown. **d.** Pseudobulk accessibility profiles, SCENIC+ predicted TFBSs (i.e. region-based eRegulon for HNF4A, CEBPA, FOXA1, ONECUT1 and TCF7L1), SCENIC+ region to gene links colored by correlation score and *Tcf7l1* and *Tbx3* expression across the zonated hepatocytes classes (from PP to PC) are shown. For the transcriptome and epigenome data, cells from 5 and 4 biological replicates were combined, respectively.

References

1. Droin, C. *et al.* Space-time logic of liver gene expression at sub-lobular scale. *Nat. Metab.* 3, 43–58 (2021).
2. Halpern, K. B. *et al.* Single-cell spatial reconstruction reveals global division of labour in the mammalian liver. *Nature* 542, 352–356 (2017).
3. Gougelet, A. *et al.* T-cell factor 4 and β-catenin chromatin occupancies pattern zonal liver metabolism in mice. *Hepatol. Baltim. Md* 59, 2344–2357 (2014).
4. Baze, M. M., Schlauch, K. & Hayes, J. P. Gene expression of the liver in response to chronic hypoxia. *Physiol. Genomics* 41, 275–288 (2010).
5. Unterberger, E. B. *et al.* Ha-ras and β-catenin oncoproteins orchestrate metabolic programs in mouse liver tumors. *Int. J. Cancer* 135, 1574–1585 (2014).
6. Boylston, W. H., DeFord, J. H. & Papaconstantinou, J. Identification of longevity-associated genes in long-lived Snell and Ames dwarf mice. *Age Dordr. Neth.* 28, 125–144 (2006).
7. Goldfarb, C. N., Karri, K., Pyatkov, M. & Waxman, D. J. Interplay Between GH-regulated, Sex-biased Liver Transcriptome and Hepatic Zonation Revealed by Single-Nucleus RNA Sequencing. *Endocrinology* 163, bqac059 (2022).
8. Meyer, R. D., Laz, E. V., Su, T. & Waxman, D. J. Male-specific hepatic Bcl6: growth hormone-induced block of transcription elongation in females and binding to target genes inversely coordinated with STAT5. *Mol. Endocrinol. Baltim. Md* 23, 1914–1926 (2009).
9. Conforto, T. L., Zhang, Y., Sherman, J. & Waxman, D. J. Impact of CUX2 on the Female Mouse Liver Transcriptome: Activation of Female-Biased Genes and Repression of Male-Biased Genes. *Mol. Cell. Biol.* 32, 4611–4627 (2012).
10. O'Brien, M. H. *et al.* Estrogen Receptor-α Suppresses Liver Carcinogenesis and Establishes Sex-Specific Gene Expression. *Cancers* 13, 2355 (2021).
11. Coste, A. de L. *et al.* Somatic mutations of the β-catenin gene are frequent in mouse and human hepatocellular carcinomas. *Proc. Natl. Acad. Sci. U. S. A.* 95, 8847–8851 (1998).
12. Ardisasmita, A. I. *et al.* A comprehensive transcriptomic comparison of hepatocyte model systems improves selection of models for experimental use. *Commun. Biol.* 5, 1–15 (2022).
13. Patwardhan, R. P. *et al.* Massively parallel functional dissection of mammalian enhancers in vivo. *Nat. Biotechnol.* 30, 265–270 (2012).
14. Inoue, F. *et al.* A systematic comparison reveals substantial differences in chromosomal versus episomal encoding of enhancer activity. *Genome Res.* 27, 38–52 (2017).
15. Ernst, J. *et al.* Genome-scale high-resolution mapping of activating and repressive nucleotides in regulatory regions. *Nat. Biotechnol.* 34, 1180–1190 (2016).
16. Klein, J. C. *et al.* A systematic evaluation of the design and context dependencies of massively parallel reporter assays. *Nat. Methods* 17, 1083–1091 (2020).
17. Smith, R. P. *et al.* Massively parallel decoding of mammalian regulatory sequences supports a flexible organizational model. *Nat. Genet.* 45, 1021–1028 (2013).
18. Davie, K. *et al.* A Single-Cell Transcriptome Atlas of the Aging Drosophila Brain. *Cell* 174, 982-998.e20 (2018).
19. Zhang, K. *et al.* A single-cell atlas of chromatin accessibility in the human genome. *Cell* 184, 5985-6001.e19 (2021).
