# Supplementary material for: Single-cell spatial multi-omics and deep learning dissect enhancer-driven gene regulatory networks in liver zonation
Source: Nat Cell Biol. 2024 Jan 5;26(1):153–67. doi: 10.1038/s41556-023-01316-4 (PMC10791584; doi:10.1038/s41556-023-01316-4)
Supplement: Supplementary file 2 — Reporting Summary [file 41556_2023_1316_MOESM2_ESM.pdf]

nature portfolio

Corresponding author(s): Stein Aerts

Last updated by author(s): Sep 12, 2023

Reporting Summary

Nature Portfolio wishes to improve the reproducibility of the work that we publish. This form provides structure for consistency and transparency in reporting. For further information on Nature Portfolio policies, see our Editorial Policies and the Editorial Policy Checklist.

Statistics

For all statistical analyses, confirm that the following items are present in the figure legend, table legend, main text, or Methods section.

n/a	Confirmed	
☐	☒	The exact sample size (*n*) for each experimental group/condition, given as a discrete number and unit of measurement
☐	☒	A statement on whether measurements were taken from distinct samples or whether the same sample was measured repeatedly
☐	☒	The statistical test(s) used AND whether they are one- or two-sided<br>*Only common tests should be described solely by name; describe more complex techniques in the Methods section.*
☐	☒	A description of all covariates tested
☐	☒	A description of any assumptions or corrections, such as tests of normality and adjustment for multiple comparisons
☐	☒	A full description of the statistical parameters including central tendency (e.g. means) or other basic estimates (e.g. regression coefficient) AND variation (e.g. standard deviation) or associated estimates of uncertainty (e.g. confidence intervals)
☐	☒	For null hypothesis testing, the test statistic (e.g. *F*, *t*, *r*) with confidence intervals, effect sizes, degrees of freedom and *P* value noted<br>*Give P values as exact values whenever suitable.*
☐	☒	For Bayesian analysis, information on the choice of priors and Markov chain Monte Carlo settings
☒	☐	For hierarchical and complex designs, identification of the appropriate level for tests and full reporting of outcomes
☐	☒	Estimates of effect sizes (e.g. Cohen's *d*, Pearson's *r*), indicating how they were calculated

Our web collection on statistics for biologists contains articles on many of the points above.

Software and code

Policy information about availability of computer code

Data collection

1. Transcriptome analysis: The 10x snRNA-seq fatsq files were processed with cellranger (v1.0.0) count function. Reads were aligned to a pre-mRNA Mus musculus reference genome (mm10), that listed each gene transcript locus as an exon, and included intronic reads in the counting (10x Genomics, see https://support.10xgenomics.com/single-cell-gene-expression/software/pipelines/latest/advanced/references#premrna). The 10x multiome fastq files were processed with cellranger-arc (v2.0.0) count function, with include introns =True option. Reads were aligned to the Mus musculus reference genome (ata-cellranger-arc-mm10-2020-A-2.0.0). 10x snRNA-seq and 10x multiome (gene expression) runs were analyzed first independently using VSN-pipelines (v0.27.0). Briefly, cells with at least 350 genes expressed and a percentage of mitochondrial reads below 10% were kept. Scanpy (v1.8.2) was run with default parameters, using the number of principal components automatically selected by VSN-Pipelines and using Leiden clustering with resolutions 0.4, 0.6 and 0.8. Hepatocyte clusters with low gene expression and high percentage of mitochondrial reads were removed, as well as doublets called with Scrublet (v0.2.3). The samples were merged, obtaining 29,798 high-quality cells, and reanalyzed with VSN-Pipelines. To correct for batch effects, we used Harmony on the selected principal components (34), using Leiden clustering with resolution 0.6, resulting in 15 clusters. The VEC and DC subpopulations were identified according to marker genes. This resulted in the identification of 14 cell types.
2. Epigenome analysis: 10x scATAC-seq samples were processed with cisTopic (v0.3.0), using the cells called by cellRanger (v1.2.0, 5,628 cells) and mm10 SCREEN regions (1,212,823 regions). For topic modelling, we used WarpLDA with default parameters, using 500 iterations and inferring models with 2, 5, 10 to 30 (by a step of 1), 35, 40, 45 and 50. This resulted in a model with 19 topics. After correcting sample effects with harmony (v1.0, applied on the scaled topic distributions), we performed Leiden clustering with resolution 0.6, obtaining 11 clusters. Gene activity was calculated by aggregating the probabilities of regions +/-10kb from the TSS (including the gene body). Cluster annotation was done based on motif enrichment, gene activity and label transfer from the annotated transcriptome with Seurat (v4.0.3, ,using cisTopic's gene activity matrix, cca as reduction and the first 10 dimensions). The labelled 10x scATAC-seq and multiome cells (annotated based on the transcriptome labels) and the scATAC-seq fragments were used as input for pycisTopic (v1.0.1.dev75+g3d3b721). Briefly, we first created pseudobulks per cell type and performed peak calling using MACS2 (v2.2.7.1, with –format BEDPE –keep-dup all –shift 73 –ext_size 146 as

parameters, as recommended for single-cell ATAC-seq data). To derive a set of consensus peaks, we used the iterative overlap peak merging procedure describe in Corces et al. (2018). First, each summit is extended a 'peak_half_width' (by default, 250bp) in each direction and then we iteratively filter out less significant peaks that overlap with a more significant one. During this procedure peaks are merged and depending on the number of peaks included into them, different processes will happen: 1) 1 peak: The original peak will be kept, 2) 2 peaks: The original peak region with the highest score will be kept and 3) 3 or more peaks: The original region with the most significant score will be taken, and all the original peak regions in this merged peak region that overlap with the significant peak region will be removed. The process is repeated with the next most significant peak (if it was not removed already) until all peaks are processed. This procedure will happen twice, first in each pseudobulk peaks, and after peak score normalization to process all peaks together. This resulted in 486,888 regions. We further filtered the data set based on the scATAC-seq quality as well, keeping cells with at least 1,000 fragments, FRiP > 0.4 and TSS enrichment > 7, resulting in 22,600 high-quality cells. Topic modelling was performed using Mallet (v2.0), using 500 iterations and models with 2 topics and from 5 to 100 by an increase of 5. Additional models between 75 and 85 (by an increase of 1) were added as we observed that the best model should be on that area based on the model selection metrics, and we selected a model with 82 topics. Batch effects between samples were corrected using harmonypy (v0.0.6) on the scaled topic distributions, and Leiden clustering with resolution 0.6 resulted in 11 clusters, corresponding to 14 cell types based on previous labelling. Drop-out imputation was performed by multiplying the region-topic and topic-cell probabilities. The imputed accessibility matrix was multiplied by 10^6. Differentially Accessible Regions (DARs) were calculated between all cell populations and specifically within hepatocytes, HSC and LSEC subgroups, using default parameters and topics were binarized using Otsu thresholding. Hepatocyte DARs and shared hepatocyte topics were curated by performing hierarchical clustering on the pseudobulk probabilities, removing a small fraction of lowly and generally accessible regions, and defining non-overlapping groups between the different gradient groups. Gene Onthology analysis was performed using GREAT (v4). We additionally run MACS2 (v2.2.7.1) bdgdiff between hepatocytes, LSEC and HSC zonated state using default parameters. The number of shared regions across mice was calculated as the regions in the shared curated topics. PycisTarget (v1.0.1.dev42+gb6707ee) was run using a custom database with the consensus regions, on DARs, binarized topics (with Otsu thresholding), curated DARs and topics and MACS2 (v2.2.7.1) bdgdiff, with and without promoters, and using pycisTarget and DEM.
3. Multiome analysis: The gene expression matrix, the imputed accessibility from pycisTopic and the motif enrichment results were used as input for SCENIC+ (v 0.1.dev411+gf4bcae5.d20220810), using only the multiome cells for eGRN inference. SCENIC+ was run with default parameters, on the complete data set and only using hepatocytes, using http://nov2020.archive.ensembl.org/ as Biomart host. Briefly, a search space of a maximum between either the boundary of the closest gene or 150 kb and a minimum of 1 kb upstream of the TSS or downstream of the end of the gene was considered for calculating region-to-gene relationships using gradient boosting machine regression. TF-to-gene relationships were calculated using gradient boosting machine regression between all TFs and all genes. Final eRegulons were constructed using the GSEA approach in which region-to-gene relationships were binarized based on gradient boosting machine regression importance scores using the 85th, 90th and 95th quantile; the top 5, 10 and 15 regions per gene and using the BASC method for binarization. Regulons between the two runs (with all cells and only hepatocytes) were merged. Gene-based and region-based regulons were scored in the relevant data sets (multiome, all scRNA-seq and scATAC-seq and spatial templates) using AUCell (v1.22.0). Regulons with positive region-to-gene relationships, at least 20 target genes and a correlation between gene-based and region-based AUC scores above 0.4 were kept, obtaining 180 high quality regulons.
4. smFISH data analysis: The completely automated imaging process per round (including water immersion generation and precise relocation of regions to image in all three dimensions) was realized by a custom python script using the scripting API of the Zeiss ZEN software (Open application development, v2023.02.27). Final image analysis was performed in ImageJ (v2.3.0/1.53f) using the Polylux tool plugin (v1.6.1) from Resolve BioSciences to examine specific Molecular CartographyTM signals. Nuclei segmentation was performed using QuPATH (v4.2.1) based on the DAPI signal, setting pixel size to 0.25, minimum area to 10, maximum area to 400, sigma to 1.7 and cell expansion 8. Data was analyzed using Seurat. Using 14 PCs, we performed Leiden clustering, resulting in 19 clusters that corresponded to 11 cell types, that were annotated based on marker gene expression.
5. Hi-C and ChIP-seq data analysis: To validate regulons, we used publicly available Hi-C and ChIP-seq data, and generated new Tbx3 ChIP-seq data. Briefly, the Hi-C data was processed using Juicer (v1.9.9), extracting values using KR for normalization by 5kb windows, and keeping only links with score > 10 and involving a bin that overlaps at least one of the consensus peaks and a TSS (+/-1000bp), resulting in 890,488 region-gene links. For the ChIP-seq data processing, reads were mapped to the mm10 genome using Bowtie2 (v2.3.5.1), peaks were called with MACS2 (v2.2.7.1, with --format BAM --gsize mm --qvalue 0.05 --nomodel --keep-dup all --call-summits –nolambda as options) bigwig files were generated using deepTools bamCoverage function (v3.5.0, with --normalizeUsing CPM --binSize 1 as parameters). Coverage on the regulon regions was obtained with deepTools computeMatrix.
6. MPRA analysis: CHEQ-seq barcodes were extracted from the plasmid and cDNA samples (read 2) using cutadapt (v1.18) with parameters with options -g TTATCATGTCTGCTCGAAGC...GATCGGCGCGCCTGCTCG --discard-untrimmed -m 17 -M 17 for the 12K libraries and g GTATCTTATCATGTCTGCTCGAAGC...GATCGGC -j 10 --discard-untrimmed -m 18 -M 18 for the 455 library and seqkit (v0.10.2), with options seq -r -p, was used to get the reverse complement sequence. Reads were filtered to keep only those with quality > 30 using fastp (v0.20.0). Reads were assigned to enhancers based on the corresponding enhancer-barcode assignments, resulting in a count matrix with number of reads per enhancer and sample. Samples were processed using DESeq2 (v1.37.6), comparing the corresponding cDNA replicates versus their plasmid samples. For the FACS fractions, since we did not extract plasmid DNA from the samples, we used the plasmid replicates from the in vivo bulk experiment. To assess enhancer activity, we used the LogFC calculated by DESeq2 (v1.37.6). To distinguish active and inactive enhancers, a Gaussian fit of the shuffled negative control values was performed with robustbase (v0.93-6), and a p-value and Benjamini–Hochberg adjusted p-value was calculated based on that Gaussian fit for all enhancers. An enhancer is considered active if its adjusted p-value is < 0.1. For the FACS experiments, FACSDiva (v9.0.1) was used.
7. ATAC-seq analysis: Adapters we removed with fastq-mcf (ea-utils v1.12) and cleaned reads were mapped to the mm10 (or hg19 for HepG2) genome using Bowtie2 (v2.3.5.1). For the FACS-ATAC experiment, a fragment count matrix was generated using the liver scATAC-seq consensus peaks using SubRead (v1.6.3). AUCell was used to assess the enrichment of the `core` signatures (general, periportal, pericentral-intermediate and pericentral) in each of the fractions, using default parameters.
8. Human data analysis: Human liver data was obtained from Zhang et al (2021). The authors labels and the scATAC-seq fragments were used as input for pycisTopic (v1.0.1.dev75+g3d3b721). Briefly, we first inferred consensus peaks as previously described, resulting in a data set with 121,593 regions and 6,366 cells. Topic modelling was performed using Mallet (v2.0), using 500 iterations and models with 2 topics and from 5 to 100 by an increase of 5. Drop-out imputation was performed by multiplying the region-topic and topic-cell probabilities. The imputed accessibility matrix was multiplied by 10^6. The mouse region-based regulons were transformed to hg38 coordinates using liftOver (https://genome.ucsc.edu/cgi-bin/hgLiftOver). The imputed accessibility matrix and the liftovered signatures were used as input for AUCell (v1.22.0) to assess regulon enrichment.

Data analysis

VSN-Pipelines as available at https://vsn-pipelines.readthedocs.io/. pycisTopic is available at https://pycistopic.readthedocs.io/. pycistarget is available at https://pycistarget.readthedocs.io/. SCENIC+ is available at https://scenicplus.readthedocs.io/. ScoMAP is available at https://github.com/aertslab/ScoMAP. Notebooks to reproduce the main figures are available at https://github.com/aertslab/Bravo_et_al_Liver.

DeepLiver is available in Zenodo (https://zenodo.org/record/8139953#) and is available in Kipoi (https://kipoi.org/docs/).

For manuscripts utilizing custom algorithms or software that are central to the research but not yet described in published literature, software must be made available to editors and reviewers. We strongly encourage code deposition in a community repository (e.g. GitHub). See the Nature Portfolio guidelines for submitting code & software for further information.

Data

Policy information about availability of data

All manuscripts must include a data availability statement. This statement should provide the following information, where applicable:

- Accession codes, unique identifiers, or web links for publicly available datasets
- A description of any restrictions on data availability
- For clinical datasets or third party data, please ensure that the statement adheres to our policy

Data generated in this manuscript (single cell RNA-seq, single cell ATAC-seq, single cell multiome, Tbx3 ChIP-seq, MPRAs and bulk ATAC-seq) is available at GEO (GSE218472). Signatures for Ras signalling, Wnt signalling pituitary response and hypoxia were obtained from Halpern et al. (2017). Single cell RNA-seq data of the mouse liver at different time points of the circadian rhythm was downloaded from GEO (GSE145197). ChIP-seq data for Hnf4a, Cebpa, Foxa1 and Onecut was downloaded from ENA (PRJEB1571). Hi-C data was obtained from GEO (GSE65126). Raw snRNA data from male and female livers was downloaded from the SRA Project PRJNA779049 (Vehicle_Female_liver: SAMN23009762 and Vehicle_Male_liver: SAMN23009760). Bulk RNA-seq data of HepG2, Hepa1-6 and AML12 was obtained from GEO (ENCFF790EGR, GSE167316, GSE146053). Data for MPRA positive controls was retrieved from ENCODE (ENCFF288HIT, ENCFF032RDN), GEO (GSE71279) and Klein et al. (2018). Saturation mutagenesis data was downloaded from https://mpra.gs.washington.edu/satMutMPRA/ and Patwardhan et al. (2012). DeepExplainer plots for each of the wild-type zonated enhancers selected for the library design in Figure 5 are available in FigShare (DOI: 10.6084/m9.figshare.24115986). DeepExplainer plots for each of the wild-type zonated enhancers selected for the library design in Figure 5 are available in FigShare (DOI: 10.6084/m9.figshare.24115986). Human liver scATAC-seq data was downloaded from GEO (GSE184462). Processed data can be explored in Scope (http://scope.aertslab.org/#/Bravo_et_al_Liver, see Supplementary Note 4) and the UCSC genome browser (https://genome.ucsc.edu/s/cbravo/Bravo_et_al_Liver, see Supplementary Note 5). Source data are provided with this study.

Human research participants

Policy information about studies involving human research participants and Sex and Gender in Research.

Reporting on sex and gender	NA
Population characteristics	NA
Recruitment	NA
Ethics oversight	NA

Note that full information on the approval of the study protocol must also be provided in the manuscript.

Field-specific reporting

Please select the one below that is the best fit for your research. If you are not sure, read the appropriate sections before making your selection.

☒ Life sciences ☐ Behavioural & social sciences ☐ Ecological, evolutionary & environmental sciences

For a reference copy of the document with all sections, see nature.com/documents/nr-reporting-summary-flat.pdf

Life sciences study design

All studies must disclose on these points even when the disclosure is negative.

Sample size	No statistical method was used to predetermine sample size. For each analysis the sample size was sufficient to derive statistically meaningful results passing multiple testing procedures.
Data exclusions	- Transcriptome analysis: 10x snRNA-seq and 10x multiome (gene expression) runs were analyzed first independently using VSN-pipelines (v0.27.0). Briefly, cells with at least 350 genes expressed and a percentage of mitochondrial reads below 10% were kept. Scanpy (v1.8.2) was run with default parameters, using the number of principal components automatically selected by VSN-Pipelines and using Leiden clustering with resolutions 0.4, 0.6 and 0.8. Hepatocyte clusters with low gene expression and high percentage of mitochondrial reads were removed, as well as doublets called with Scrublet (v0.2.3). The samples were merged, obtaining 29,798 high-quality cells, and reanalyzed with VSN-Pipelines<br>- Epigenome analysis: We filtered the data set based on the scATAC-seq quality, keeping cells with at least 1,000 fragments, FRiP > 0.4 and TSS enrichment > 7, resulting in 22,600 high-quality cells.
Replication	For the single-cell experiments we performed at least two experiments per technique (snRNA-seq, snATAC-seq or single cell multiomics). For the smFISH we performed three replicates. For the MPRA experiments we performed at least two replicates per condition (system [HepG2, AML12, Mouse] and library). For the luciferase experiments we performed tfour replicates per condition. In all experiments, results between

	experiments were similar.
Randomization	Not relevant, in each experiment groups were independent.
Blinding	Blinding was not relevant to the study as the computational analyses are fully reproducible.

Reporting for specific materials, systems and methods

We require information from authors about some types of materials, experimental systems and methods used in many studies. Here, indicate whether each material, system or method listed is relevant to your study. If you are not sure if a list item applies to your research, read the appropriate section before selecting a response.

Materials & experimental systems

n/a	Involved in the study	
☐	☒	Antibodies
☐	☒	Eukaryotic cell lines
☒	☐	Palaeontology and archaeology
☐	☒	Animals and other organisms
☒	☐	Clinical data
☒	☐	Dual use research of concern

Methods

n/a	Involved in the study	
☐	☒	ChIP-seq
☐	☒	Flow cytometry
☒	☐	MRI-based neuroimaging

Antibodies

Antibodies used	Cells used for the FACS experiments were stained with the following antibodies (BioLegend) at a dilution of 1:300: PE anti-mouse/human CD324 E-cadherin (catalogue no. 147304) and APC anti-mouse CD73 (catalogue no. 127210). For ChIP-seq, Bethyl rabbit anti-TBX3 Antibody was used (Sanbio A303-098A, lot number #`1).
Validation	More information about the antibodies is provided at https://www.biolegend.com/en-us/products/pe-anti-mouse-human-cd324-e-cadherin-antibody-9276 and https://www.biolegend.com/en-us/products/apc-anti-mouse-cd73-antibody-7893. For the ChIP-seq antibody, more information is available at https://www.sanbio.nl/catalog/product/view/id/569891 and https://www.thermofisher.com/antibody/product/TBX3-Antibody-Polyclonal/A303-098A. The TBX3 antibody was tested for western blot and immunoprecipitation, giving similar results as the rabbit anti-TBX3 antibody BL8058, which recognizes a dowstream epitope (see website).

Eukaryotic cell lines

Policy information about cell lines and Sex and Gender in Research

Cell line source(s)	The HepG2, Hepa1-6 and AML12 cell lines were purchased from ATCC (HB-8065, CRK-1830 and CRL-2254, respectively).
Authentication	Cell lines were not authentificated in-house, as they were obtained from a commercial source.
Mycoplasma contamination	HepG2, Hepa1-6 and AML12 cells tested negative for mycoplasma contamination.
Commonly misidentified lines (See ICLAC register)	We confirm that none of the cell lines used in this study is a Commonly Misidentified line.

Animals and other research organisms

Policy information about studies involving animals; ARRIVE guidelines recommended for reporting animal research, and Sex and Gender in Research

Laboratory animals	Adult male mice (8 to 10 weeks old) were used in this study. All mice were C57BL/6JaxCrl except for mouse 1 in the single-cell experiments, which was Crl:CD-1. Mice were maintained under standard housing conditions, with continuous access to food and water; except for mice 4 and 5 in the single-cell experiments, for which food was removed approximately 10 hours before the experiments.
Wild animals	No wild animals were used in this study.
Reporting on sex	All mice used in this study were male. We used publically available data on female and male livers (Goldfarb et al. 2022) to assess the relevance of the eGRN networks inferred from male liver data.
Field-collected samples	No field samples were collected.
Ethics oversight	All animal experiments were conducted according to the KU Leuven ethical guidelines and approved by the KU Leuven Ethical

Ethics oversight	Committee for Animal Experimentation (approved protocol number ECD P007/2021).

Note that full information on the approval of the study protocol must also be provided in the manuscript.

ChIP-seq

Data deposition

☒ Confirm that both raw and final processed data have been deposited in a public database such as GEO.

☒ Confirm that you have deposited or provided access to graph files (e.g. BED files) for the called peaks.

Data access links<br>*May remain private before publication.*	Tbx3 ChIP-seq raw data, processed bigwig files and MACS called peaks are available at GEO (GEO218472).
Files in database submission	Fastq, bigwig and narrowPeak files.
Genome browser session<br>(e.g. UCSC)	Data can be visualized at https://genome.ucsc.edu/s/cbravo/Bravo_et_al_Liver

Methodology

Replicates	Only 1 replicate per experiment (Tbx3 ChIP-seq and input) was performed
Sequencing depth	Libraries with fragment size of 300-500bp were sequenced using NextSeq2000 (paired end, 50bp per reads). In the Tbx3 ChIP sample, 74,325,203 reads were sequenced, out of which 93.1% were properly paired. In the input ChIP sample, 370,359,282 reads were sequenced, out of which 93.63% were properly paired.
Antibodies	Bethyl rabbit anti-TBX3 Ab (Sanbio A303-098A).
Peak calling parameters	Peaks were called with MACS2 (v2.2.7.1, with --format BAM --gsize mm --qvalue 0.01 --call-summits as options).
Data quality	Using MACS2, we identified 23,951 peaks (q-value < 0.01), out of which 19,812 overlap with regions accessible in the mouse liver. Motif enrichment was performed using pycisTarget with default parameters on the top 1,000 ChIP-seq regions, detecting the Tbx3 motif as expected.
Software	Reads were mapped to the mm10 genome using Bowtie2 (v2.3.5.1), peaks were called with MACS2 (v2.2.7.1, with --format BAM --gsize mm --qvalue 0.01 --call-summits as options) and bigwig files were generated using deepTools bamCoverage function (v3.5.0, with --normalizeUsing CPM --binSize 1 as parameters).

Flow Cytometry

Plots

Confirm that:

☒ The axis labels state the marker and fluorochrome used (e.g. CD4-FITC).

☒ The axis scales are clearly visible. Include numbers along axes only for bottom left plot of group (a 'group' is an analysis of identical markers).

☒ All plots are contour plots with outliers or pseudocolor plots.

☒ A numerical value for number of cells or percentage (with statistics) is provided.

Methodology

Sample preparation	FACS MPRA in vivo: For intrahepatic delivery of the liver MPRA libraries, 8 to 10-week-old mice were secured and hydrodynamically injected with 20 µg of the libraries via the lateral tail vein. All libraries were diluted in sterile filtered 0.9% NaCl, and the total volume was adjusted to 10% (in mL) of the total body weight (in grams). 48 hours post-injection, mice were anesthetized by intraperitoneal injection of sodium pentobarbital (Nembutal, 50 mg/kg). Livers were perfused for 5 minutes with 40 mL of perfusion medium SC-1 (8 g/L Nacl, 400 mg/L KCl, 75.5 mg/L NaH2PO4, 120.5 mg/L Na2HPO4, 2,38 g/L HEPES, 350 mg/L NaHCO3, 190 mg/L EGTA, 900 mg/L D-(+)-Glucose, 1.2 mL Phenol Red solution) to remove the blood, followed by perfusion with 40 mL of SC-2 medium (8 g/L Nacl, 400 mg/L KCl, 75.5 mg/L NaH2PO4, 120.5 mg/L Na2HPO4, 2,38 g/L HEPES, 350 mg/L NaHCO3, 560 mg/L CaCl2.2H2O, 1.2 mL Phenol Red solution) containing 10 mg of collagenase P (Merck) for 5 minutes. Each lobe was dissected off and minced into small pieces in a beaker containing 39 mL SC-2 supplemented with 1 mL DNase I (Sigma-Aldrich) and 20 mg collagenase P, followed by rotating incubation for 15 min at 37°C. Hepatocytes were centrifuged for 2 min at 50 g, washed with PBS, centrifuged again for 2 min at 50 g, resuspended in 3 ml Hoechst buffer (DMEM + 10% FBS + 10 mM HEPES) and filtered through a 70 µm strainer. The protocol for hepatocyte staining was adapted from Ben-Moshe et al. (2019)61. After counting the cells on a LUNA cell counter, the concentration was adjusted to 5 million cells in 1 mL of Hoechst buffer. To determine the ploidy of hepatocytes, DNA was stained with Hoechst (Thermo Fisher Scientific) (15 µg/mL). Reserpine (5 µM) was also supplemented to prevent Hoechst expulsion from the cells. Cells were incubated for 30 min at 37°C. Hepatocytes were centrifuged for 5 min at 1,000 rpm at 4°C and the supernatant was discarded. Cells were resuspended in cold PBS in a concentration of 1 million cells in 100 µL. After spinning down (1,000 rpm for 5 min at 4°C), cells were resuspended in FACS buffer (2 mM EDTA, pH 8, and 0.5% BSA in 1x PBS) at a concentration of 1

million cells in 100 µL. Cells were stained with the following antibodies (BioLegend) at a dilution of 1:300: PE anti-mouse/human CD324 E-cadherin (catalogue no. 147304) and APC anti-mouse CD73 (catalogue no. 127210). FcX blocking solution (BioLegend catalogue no. 101319) was added at a dilution of 1:50.
FACS ATAC-seq: Hepatocytes were isolated and stained as described in the FACS MPRA section, with minor modifications. Cells were additionally stained with Alexa Fluor 488 Zombie Green (BioLegend) to enable the detection of viable cells by FACS. Zombie Green was added at a dilution of 1:500 and cells were kept in a rotator in the dark at room temperature for 15 min.

Instrument	Cells were sorted by FACS-Aria-Fusion (BD Biosciences) using a 100 µm nozzle.
Software	FACSDiva (v9.0.1)
Cell population abundance	OmniATAC-seq was performed in the FAC-sorted fractions. AUCell was used to assess the enrichment of the \`core\` signatures (general, periportal, pericentral-intermediate and pericentral) in each of the fractions, using default parameters.
Gating strategy	FACS MPRA in vivo: FSC-A and SSC-A were used for hepatocytes size selection. Cells containing the library were selected based on GFP. Tetraploid hepatocytes were selected based on Hoechst stain. CD73 and Ecad were used to select hepatocytes bins along the porto-central axis, obtaining 100,000-200,000 cells per bin.<br>FACS ATAC-seq: FSC-A and SSC-A were used for hepatocytes size selection. Viable cells were selected based on the Zombie Green signal. Tetraploid hepatocytes were selected based on Hoechst stain. CD73 and Ecad were used to select hepatocytes bins along the porto-central axis, obtaining 20,000-50,000 cells per bin.

☒ Tick this box to confirm that a figure exemplifying the gating strategy is provided in the Supplementary Information.
